# Quasi-experimental trial of diabetes Self-Management Automated and Real-Time Telephonic Support (SMARTSteps) in a Medicaid managed care plan: study protocol

**DOI:** 10.1186/1472-6963-12-22

**Published:** 2012-01-26

**Authors:** Neda Ratanawongsa, Margaret A Handley, Judy Quan, Urmimala Sarkar, Kelly Pfeifer, Catalina Soria, Dean Schillinger

**Affiliations:** 1 General Internal Medicine and UCSF Center for Vulnerable Populations at San Francisco General Hospital and Trauma Center, University of California, San Francisco, CA, 1001 Potrero Avenue, Box 1364, San Francisco, CA 94110, USA; 2 Department of Epidemiology and Biostatistics, Division of Preventive Medicine and Public Health, University of California, San Francisco, CA, 185 Berry Street, Lobby 5, Suite 5700, San Francisco, CA 94107, USA; 3 San Francisco Health Plan, 201 3rd Street, 7th Floor, San Francisco, CA 94103, USA; 4 California Diabetes Program, California Department of Public Health, PO Box 997377, MS 7211, Sacramento, CA 95899-7377, USA

## Abstract

**Background:**

Health information technology can enhance self-management and quality of life for patients with chronic disease and overcome healthcare barriers for patients with limited English proficiency. After a randomized controlled trial of a multilingual automated telephone self-management support program (ATSM) improved patient-centered dimensions of diabetes care in safety net clinics, we collaborated with a nonprofit Medicaid managed care plan to translate research into practice, offering ATSM as a covered benefit and augmenting ATSM to promote medication activation. This paper describes the protocol of the Self-Management Automated and Real-Time Telephonic Support Project (SMARTSteps).

**Methods/Design:**

This controlled quasi-experimental trial used a wait-list variant of a stepped wedge design to enroll 362 adult health plan members with diabetes who speak English, Cantonese, or Spanish and receive care at 4 publicly-funded clinics. Through language-stratified randomization, participants were assigned to four intervention statuses: SMARTSteps-ONLY, SMARTSteps-PLUS, or wait-list for either intervention. In addition to usual primary care, intervention participants received 27 weekly calls in their preferred language with rotating queries and response-triggered education about self-care, medication adherence, safety concerns, psychological issues, and preventive services. Health coaches from the health plan called patients with out-of-range responses for collaborative goal setting and action planning. SMARTSteps-PLUS also included health coach calls to promote medication activation, adherence and intensification, if triggered by ATSM-reported non-adherence, refill non-adherence from pharmacy claims, or suboptimal cardiometabolic indicators. Wait-list patients crossed-over to SMARTSteps-ONLY or -PLUS at 6 months. For participants who agreed to structured telephone interviews at baseline and 6 months (n = 252), primary outcomes will be changes in quality of life and functional status with secondary outcomes of 6-month changes in self-management behaviors/efficacy and patient-centered processes of care. We will also evaluate 6-month changes in cardiometabolic (HbA1c, blood pressure, and LDL) and utilization indicators for all participants.

**Discussion:**

Outcomes will provide evidence regarding real-world implementation of ATSM within a Medicaid managed care plan serving safety net settings. The evaluation trial will provide insight into translating and scaling up health information technology interventions for linguistically and culturally diverse vulnerable populations with chronic disease.

**Trial Registration:**

ClinicalTrials.gov: NCT00683020

## Background

As health systems and public health agencies grapple with the rising burden of diabetes, they face significant challenges with the persistent and widening disparities in the prevalence, quality of care, and clinical outcomes across such factors as race/ethnicity, language, health literacy, and socioeconomic status [1-3]. Patient-centered, culturally concordant care is a cornerstone for reducing disparities, but vulnerable populations with limited health literacy and limited English proficiency face barriers to access and communication that place them at risk for suboptimal treatment management and poor health outcomes [4-13].

Self-management support programs are a critical component of chronic disease care delivery that can improve outcomes by providing individualized assessment, collaborative goal-setting, skills enhancement, follow-up, and access to resources and continuity of care [14-16]. Despite the proven benefits of self-management support and evidence that vulnerable populations desire these interventions [17], the translational gap between research and practice may be particularly wide in safety net settings, which disproportionately care for these patients [18,19]. Providing self-management support is resource-intensive, requiring re-training of staff and organizational change, investments in information technology, and tailoring programs to engage and serve diverse populations [20-22]. Thus, traditional self-management support approaches often do not reach significant and growing segments of the chronic disease population, such as the uninsured or publicly-insured or those with communication barriers [1,6,17,23-25]. Given the documented challenges with translating research into real-world practice, practical implementation and evaluation research is needed in settings where vulnerable populations receive care [4,26-31].

Patient-facing health information technology (HIT) holds promise to increase access to self-management support and enhance health outcomes for vulnerable populations in safety net settings [32-34]. Automated telephone self-management support (ATSM) employs phone technology to provide surveillance and education and to prioritize further care management efforts for those most in need [32]. ATSM can be used to promote collaborative goal-setting through behavioral action plans, by which patients set and achieve short-term goals to improve their self-management [35]. ATSM can also provide individualized assessment, skills enhancement, live follow-up and support from health educators or coaches, access to community resources, and continuity of clinical care [32,33]. The Institute of Medicine has highlighted ATSM as an exemplary strategy in its National Action Plan for Health Literacy [36].

The Improving Diabetes Efforts Across Language and Literacy (IDEALL) study - a randomized controlled trial of ATSM among English-, Spanish-, and Chinese-speaking patients with poorly controlled diabetes - demonstrated high engagement with ATSM, particularly among participants with limited health literacy and limited English proficiency [32]. Compared with patients receiving usual care or group medical visits, patients exposed to ATSM had greater improvements in their experiences of chronic illness care, self-management behavior, fewer bed days per month, less interference in their daily activities, with a cost utility for functional outcomes comparable to other diabetes prevention and treatment interventions [33,37]. We hypothesized that ATSM could be augmented by harnessing pharmacy claims and clinical registry data, allowing health coaches to identify patient non-adherence and missed opportunities for medication intensification and conduct patient-centered counseling outside of the time-constrained office visit [38,39]. Given these potential benefits, the San Francisco Health Plan (SFHP) - a non-profit Medicaid managed care plan serving a diverse, low-income population - approached our research team at the University of California, San Francisco (UCSF) Center for Vulnerable Populations to evaluate the implementation of ATSM as a covered health benefit for a subset of its members with diabetes and add an augmented intervention harnessing medication claims to promote medication adherence and intensification.

This paper presents a description of the study design and recruitment results for the Self-Management Automated and Real-Time Telephonic Support Study (SMARTSteps/Pasos Positivos/ 明智進步計劃), a controlled quasi-experimental evaluation study to improve diabetes quality of life, self-management, and clinical outcomes with two variants of language-concordant ATSM intervention implemented by a Medicaid managed care plan serving a low-income, ethnically diverse urban population. We hypothesized that intervention participants would demonstrate greater improvements in diabetes self-management, patient-centered outcomes, and cardiometabolic outcomes compared to wait-list controls and that improvements would be greatest for patients exposed to ATSM with an enhanced medication activation communication strategy triggered by pharmacy claims and self-reported medication non-adherence.

## Methods/Design

### Study aims

Among ethnically and linguistically diverse persons with diabetes receiving care in a primary care safety net system:

*Primary Aim: *To investigate differences in 6-month changes in patient-centered outcomes including quality of life and functional status (SF-12 and number of days spent in bed due to illness), comparing patients exposed to ATSM with wait-list controls and comparing patients exposed to ATSM (SMARTSteps-ONLY) with ATSM augmented by medication adherence and intensification (SMARTSteps-PLUS).

*Secondary Aims: *To investigate differences in 6-month changes in diabetes self-management behaviors and self-efficacy, patient-centered processes of care, cardiometabolic outcomes (HbA1c, blood pressure, and LDL), receipt of recommended health services, and healthcare utilization, comparing patients exposed to ATSM with wait-list controls and comparing patients exposed to ATSM (SMARTSteps-ONLY) with ATSM augmented by medication adherence and intensification (SMARTSteps-PLUS).

### Study Design

**Figure 1:**
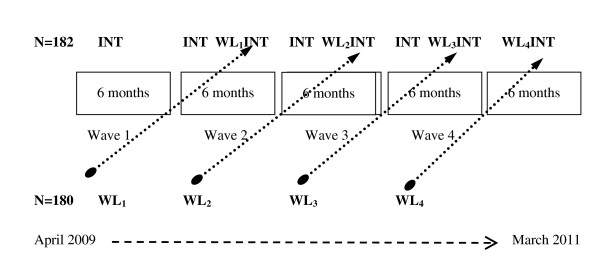
**Quasi-experimental evaluation trial design of SMARTSteps, a language-concordant automated telephone diabetes self-management health plan intervention**. **INT **= Intervention Group, **WL **= Wait-list Group, **WLINT **= Wait-list Group Turned Intervention (after 6 months). Represented in the figure are 182 Medicaid managed care plan members who were randomized to intervention and 180 who were randomized to wait-list over the entire study period. Each 6-month enrollment phase (the boxes identified as 'waves') had patients going directly into an intervention arm (INT) or wait-list for 6 months (WL). Each wave of wait-list patients was then crossed-over into intervention after 6 months (WLINT). The dots represent the cross-over for individuals on wait-list (WL) into the active intervention arm (WLINT).

The study was a controlled quasi-experimental evaluation trial with a wait-list variant of a stepped wedge design (see Figure 1), which has been described previously in the implementation science literature [40-42]. This design is characterized by staggered introduction of an intervention over time, cross-over of individual patients from control to intervention arm at designated times, and multiple data collection points. A stepped wedge design allows for practical allocation of intervention resources over time, with introduction of the intervention in stages, such that all members receive the intervention eventually, while still retaining elements of a controlled trial [40]. Compared with a randomized clinical trial, or a simple before and after design, the stepped wedge design was more acceptable to SFHP, which felt it would be unethical to deny some members ATSM as a covered benefit while offering it to others, given prior evidence of ATSM's positive impact on health outcomes. This design also allowed SFHP to scale up recruitment and implementation in a controlled fashion, with less intensive staffing than required if offering the intervention to all eligible members at project initiation (as in a cluster randomized design). Finally, conducting four recruitment waves over 24 months allowed for collection of intervention and control data across multiple waves, allowing analysis to take into account possible variations over time [40].

### Study Setting

The study sample was drawn from SFHP members who received primary care at one of four publicly-funded clinics in the Community Health Network of San Francisco (CHNSF). SFHP is a non-profit Medicaid managed care plan created in 1994 to provide high quality medical care to the largest possible number of low-income San Francisco residents. In addition to managing Medicaid benefits, SFHP administers "Healthy Workers," an insurance program for people working as in-home support service providers for elderly or disabled people in San Francisco. At the time SFHP contacted the UCSF investigative team in 2008, it managed the care of approximately 50,000 enrollees and served an ethnically diverse population comprised of 48% Asian-Americans, 26% Latinos, 12% African-Americans; 60% of their membership were non-English speaking.

CHNSF is the integrated healthcare delivery system of the San Francisco Department of Public Health and part of the UCSF San Francisco Bay Area Collaborative Research Network, one of the longest standing U.S. practice-based research networks (http://familymedicine.medschool.ucsf.edu/research/research_programs/crn/crn.aspx). CHNSF is comprised of 12 community health centers and hospital-based clinics centers affiliated with the county's acute care facility, San Francisco General Hospital. CHNSF maintains a single electronic medical information system with unique patient identifiers across the clinical sites and a clinical registry that integrates patient data such as diagnoses, vital signs, and laboratory data.

### Study sample

#### Eligibility criteria

Eligible participants were characterized by the following: SFHP membership; ≥1 primary care clinic visit in the preceding 24 months at one of our designated clinics; age 18 or above; a diagnosis of diabetes (type 1 or 2); English-, Cantonese-, or Spanish-speaking; access to a touch-tone phone; and plans to remain in the region during the evaluation period (12 months). Patients who were pregnant or unable to provide verbal consent were excluded. The diagnosis of diabetes was assessed in two ways. First, we searched for SFHP members in the CHNSF electronic diabetes registry. Second, we searched for members with SFHP claims data consistent with diabetes (ICD-9 codes for utilization and pharmacy claims for oral or injectable glycemic medications or glucose self-monitoring supplies). If members were identified through SFHP claims data but were not found in the registry, we validated the diabetes diagnosis by reviewing electronic health records for fasting glucose ≥ 126 mg/dl and/or clinician-documented diagnosis of diabetes based on HbA1c ≥ 7% [43]. During the course of the evaluation trial, the American Diabetes Association revised diagnostic criteria to include HbA1c ≥6.5% [44], but we did not modify our eligibility criteria to avoid introducing selection bias and because evidence suggests a variable delay in the adoption of guideline criteria by practicing providers [45,46].

#### Sample recruitment procedures

SFHP mailed to potentially eligible members' homes and clinicians' offices SMARTSteps post cards describing this new benefit in English, Spanish, and Cantonese and offering an SFHP number to call for information. SFHP enrollment workers fluent in these three languages also actively recruited potentially eligible health plan members through scripted telephone calls. The enrollment workers confirmed eligibility for SMARTSteps by phone and offered $25 gift cards as incentives for participation. All eligible members who reported interest in SMARTSteps were then randomized by SFHP to one of four intervention statuses (described below) regardless of interest in the UCSF evaluation.

SFHP workers also assessed participant willingness to complete evaluation interviews administered by UCSF staff. Those members willing to participate in the interviews were then telephoned by UCSF research assistants to obtain verbal consent for participation in the evaluation interviews and were offered $50 gift cards as incentives for each interview completed (up to $150).

#### Ethics approval

The Committee on Human Research at the University of California, San Francisco (H9894-32044-01) approved collection of interview data by UCSF from consenting participants after obtaining verbal consent. Because all recruitment and implementation was conducted by SFHP by phone, it was felt that requiring a separate visit to obtain written consent at UCSF or the primary care clinic sites reduce participation, leading to selection bias, and disrupt clinic workflow. Because the interviews offered minimal risk, the Committee approved verbal telephone consent for these interviews. In addition, ethics approval included waiver of informed consent to abstract and analyze CHNSF clinical data from the CHNSF electronic health record, CHNSF diabetes registry, SFHP claims data, and SFHP health coaching data for all SFHP members eligible for SMARTSteps.

### Study arms

All eligible SFHP members interested in the SMARTSteps program were assigned by SFHP through language-stratified randomization to one of four intervention statuses: SMARTSteps-ONLY, SMARTSteps-PLUS, wait-list for SMARTSteps-ONLY, or wait-list for SMARTSteps-PLUS.

#### Wait-list Control

Those randomized to the wait-list continued to receive usual care through their clinics, as well as all existing SFHP benefits (reminders and incentives for receipt of recommended health services, including laboratory testing, eye and foot examination, and influenza vaccination) [43,47]. At the end of the 6-month wait-list period, each participant "crossed-over" to begin SMARTSteps-ONLY or SMARTSteps-PLUS, depending on initial randomization arm.

#### SMARTSteps-ONLY

Participants randomized to SMARTSteps-ONLY received the ATSM intervention within 2 weeks. Developed with extensive input from patients to be sensitive to literacy, language, and culture in the target populations [32], this ATSM system provided 27 weeks of 8-12 minute weekly calls in English, Cantonese, or Spanish. Patients specified the weekday and time convenient for their schedules or called toll-free into the system if they missed their scheduled call. The content consisted of rotating sets of queries about self-care (such as diet, exercise, and medication adherence), psychosocial issues (such as depressive symptoms), and access to preventive services (such as eye care). Patients responded via touch-tone commands, and based on their answers, patients heard automated health education messages in the form of narratives.

Patients answering "out-of-range" on an item or selecting a "call back" option received a telephone call within 3 days from a language-concordant SFHP health coach. This system was designed to promote health coach efficiency and effectiveness by focusing outreach calls to patients needing further support based on ATSM responses. Health coaches provided education and engaged in collaborative goal-setting to form patient-centered action plans, a core process for self-management support by which patients set short-term goals to improve their self-management [20,35].

SFHP used a health coach for responding to ATSM triggers, rather than a nurse practitioner care manager as was done in the IDEALL trial [33]. Health coaches - SFHP employees without a medical background or postgraduate training - conducted phone calls under the supervision of a registered nurse care manager. SFHP registered nurse supervisors and UCSF study physicians taught health coaches about diabetes and trained them in behavior change counseling and overcoming barriers to health communication. UCSF staff also guided the development of written protocols and scripts to respond to potential ATSM call triggers, such as assessing hypoglycemia symptoms or causes.

Health coaches documented the content of their phone interactions, including patient-generated action plans and whether they were achieved, using an SFHP care management database system. SFHP health coaches also contacted the designated clinic contact and/or the primary care provider (PCP) using standardized templates for patients with pre-specified safety issues (such as a new medical symptom) or access concerns (such as need for refills or appointments). Non-urgent issues were communicated by email or fax, while urgent issues were also conveyed by phone.

Patients who indicated on their ATSM responses that they missed their diabetes, blood pressure, or cholesterol medications for 3-7 days in the previous week received calls from health coaches to troubleshoot barriers to adherence. These calls were only triggered by patient self-disclosure in response to ATSM queries, unlike adherence counseling calls triggered for SMARTSteps-PLUS participants described below.

#### SMARTSteps-PLUS

The goal of the SMARTSteps-PLUS intervention was to detect and intervene for participants whose medication treatment was suboptimal, either because of non-adherence or potential missed opportunities to intensify their regimens. Because SFHP does not have prescribing authorization for its members, the intervention focused on enhancing medication regimens and adherence through collaborative goal-setting with patients and feedback to PCPs. Participants randomized to SMARTSteps-PLUS immediately received the ATSM intervention described above, as well as medication activation and intensification coaching triggered by refill non-adherence or suboptimal achievement of cardiometabolic treatment goals. Based on review of evidence-based guidelines [43], SFHP and UCSF collaborated to develop a protocol to improve medication adherence and promote patient-centered intensification by harnessing electronically available data from two additional sources: monthly SFHP pharmacy claims and clinical laboratory and blood pressure data from the electronic health records of the CHNSF.

**Figure 2:**
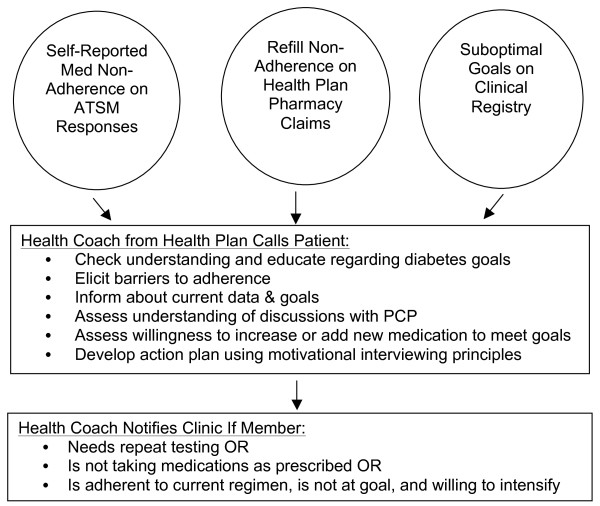
**SMARTSteps-PLUS protocol for automated telephone self-management (ATSM) with enhanced medication adherence and intensification counseling**.

The medication activation protocol targeted 3 groups of patients within the SMARTSteps-PLUS arm (see Figure 2):

1. Patients who indicated in response to weekly ATSM queries that they missed 3-7 days of medications in the last week.

2. Patients with a 15-day to 6-month gap in refilling specific cardiometabolic prescriptions: oral hypoglycemic, insulin, anti-hypertensives (including ACE inhibitors and angiotension-receptor blockers), cholesterol-lowering medications, or glucose testing strips (for patients receiving insulin or sulfonylureas), based on pharmacy claims data. We chose a lower limit of 15-days to account for potential delays in SFHP receipt of pharmacy claims and an upper limit of 6 months in case healthcare providers actively discontinued these medications.

3. Patients with suboptimal achievement of cardiometabolic goals [43]:

• Systolic blood pressure ≥ 130 mmHg at last recorded check or no measurement in clinical registry within preceding 6 months

• Diastolic blood pressure ≥ 80 mmHg at last recorded check or no measurement in clinical registry within preceding 6 months

• Hemoglobin A1c (HbA1c) > 7.0% on last measurement or no measurement in clinical registry within preceding 6 months

• Low-density lipoprotein (LDL) > 100 mg/dL on last measurement or no measurement in clinical registry within preceding 12 months

• Urinary albumin/creatinine > 30 mcg/mg for patients who were not prescribed or late to refill an ACE inhibitor or angiotensin-receptor blocker

An individual SMARTSteps-PLUS participant could fall into none, one, or more than one of these groups at any given time. SFHP queried the pharmacy claims and clinical registry data monthly to provide health coaches with a list of SMARTSteps-PLUS participants to be called.

For patients with evidence of non-adherence, SFHP health coaches were trained with specific written protocols and scripts to promote self-disclosure of medication non-adherence and troubleshoot barriers such as confusion about doses or frequency of medications, forgetfulness, concerns about side effects, or health beliefs (see Additional File 1). For patients who had not had cardiometabolic measurements within guideline-concordant timeframes, health coaches counseled patients about the reasons for these measures, encouraged them to talk with their providers, and notified PCPs of patients willing to obtain these tests. For patients with suboptimal achievement of treatment goals, health coaches were trained to counsel patients about their blood pressure or lab values and assess their willingness to discuss with their PCPs the possibility of taking more medication. Health coaches also encouraged patients to undergo potential lifestyle changes. Based on their discussions, health coaches engaged patients in collaborative goal-setting and action planning [20,35]. Health coaches then notified the clinic contacts and/or PCPs about interactions with any patients who needed laboratory or blood pressure assessments, reported barriers to medication adherence, or indicated a willingness to discuss medication intensification.

### Study integrity

The study design conforms to CONSORT statement recommendations for randomized trials of non-pharmacologic treatment [48]. Randomization to intervention groups was conducted by SFHP before assessment of willingness to participate in UCSF evaluation interviews to permit analysis of primary outcomes. Intervention arm assignment was conducted using a 4 × 4 blocked randomized design, with the participant as the unit of randomization. This randomization was completed for each of the three study languages. Research assistants administering the evaluation interviews were blinded to group allocation. The intervention protocol is documented and filed on secure servers. Data generated during ATSM and health coach calls is stored in the ATSM and SFHP database systems, respectively. All analyses will be intent-to-treat.

### Measurement

#### Data Collection

Table 1 lists study outcomes.

**Table 1:** Outcomes for SMARTSteps, a quasi-experimental evaluation trial of a language-concordant automated telephone diabetes self-management health plan intervention

Variable	Instrument
**Primary outcome variables**	

Functional status	SF-12 [51] Days confined to bed due to illness

**Secondary outcome variables**	

Diabetes self-management behaviors	Summary of Diabetes Self-Care Activities (SDSCA) measure [52]

Self-reported medication adherence	Summary of Diabetes Self-Care Activities (SDSCA) measure [52]

Diabetes self-efficacy	Patient self-management scale derived from questionnaire used in the Diabetes Quality Improvement Project [54]

Patient-centeredness of care	Patient Assessment of Care for Chronic Conditions (PACIC) [55] Interpersonal processes of care (IPC) [56]

Glycemic control	Hemoglobin A1c

Blood pressure control	Systolic and diastolic blood pressure

Cholesterol control	Low-density lipoprotein

Quality of care	Proportions receiving hemoglobin A1c and blood pressure measurement within 6 months [43,47]

	Proportions receiving LDL and microalbumin/creatinine measurement within 12 months [43,47]

	Proportion receiving retinal examination within 12 months [43,47]

	Proportion receiving influenza and pneumococcal vaccination [43,47]

Utilization	Emergency department Hospitalization

For participants consenting to the UCSF evaluation trial, measures of all primary and secondary patient-reported outcomes were derived from structured interviews conducted in English, Spanish or Cantonese. For Spanish and Cantonese interviews, survey questions were translated and back-translated in their respective languages. Interviews were administered using computer-assisted telephone interview (CATI) software or paper survey, with responses later entered into the CATI-created database. Consenting participants completed baseline interviews within 2 weeks of being randomized, prior to the first ATSM call. SMARTSteps-ONLY and SMARTSteps-PLUS participants completed the 6-month follow-up interviews after the conclusion of their ATSM intervention, and wait-list participants completed the 6-month follow-up interviews before the start of their ATSM calls. Wait-list groups received an additional 6-month follow-up interview (12 months after enrollment) after completion of intervention period. Participants received a $50 gift card for a pharmacy store at the completion of each interview.

For all eligible SFHP members, we collected socio-demographic variables and cardiometabolic outcomes data using monthly downloads of SFHP claims data and CHNSF clinical registry data. At the conclusion of the project, we will obtain SFHP administrative and claims data for utilization outcomes.

#### Socio-demographic variables

Self-reported socio-demographic variables included age, gender, race/ethnicity, language, English proficiency, years since immigration to the U.S., marital status, educational attainment, employment status, annual household income, self-reported health literacy [49,50], and duration of diabetes. We obtained SFHP administrative and CHNSF data regarding age and gender. Preferred language is captured in both SFHP and CHNSF data, and where this preference was discrepant, we used the language reported to SFHP enrollment workers.

#### Primary and Secondary Patient-Reported Outcomes

The primary outcome variables are physical and mental functional status (SF-12) and the number of days spent in bed due to illness [51]. Secondary outcomes include diabetes self-efficacy and self-management behavior, as well as medication adherence in the preceding 7 days [52-54]. Secondary outcomes also include patient perspectives on the structure of their care, as measured by two instruments which have been translated and validated for our population in our prior work: Patient Assessment of Care for Chronic Conditions (PACIC) and interpersonal processes of care (IPC) [33,55,56].

#### Cardiometabolic Outcomes

These secondary outcomes will be analyzed on the larger set of SFHP participants randomized in the trial (N = 362), regardless of whether or not they participated in the evaluation interviews. Cardiometabolic outcomes include measures of HbA1C, LDL, and blood pressure obtained by clinical providers. Data will be derived from CHNSF electronic health record, the clinical registry database, and paper chart review. Values will be used at baseline only if obtained within one year before program enrollment. Follow-up laboratory and blood pressure values will be included only if obtained within 90 days before or after the 6-month and 12-month follow-up dates.

#### Care Delivery Processes and Utilization

Finally, for all eligible participants, we will use SFHP administrative claims data to examine measures of care delivery processes: proportion receiving blood pressure checks, laboratory testing, retinal examinations, influenza vaccination, and pneumococcal vaccination at guideline-concordant frequencies [43]. We will also examine utilization, including the frequency of emergency department visits and hospitalizations.

#### Program Engagement and Satisfaction

Using data from the ATSM system, we will assess patient engagement as measured by exposure intensity (defined as the proportion of the 27 weeks of exposure, participants replied to the weekly calls) and the proportion of weekly calls that are determined to be complete (calls answered and responses to 100% of queries), partially complete (call answered and responses to < 50% of queries), and incomplete (call answered but responses to 0% of queries) [32]. In addition, using data from the SFHP health coach database, we will analyze at the participant level the number of health coach calls that were triggered by ATSM responses; the number of calls attempted and/or completed by health coaches; and the number of action plans created and/or achieved, as reported by patients and documented in the database. We will conduct fidelity assessments to examine potential differential engagement across languages, clinics and study arms. Finally, we will assess satisfaction and perceived usefulness of the intervention as reported during evaluation interviews at 6- and 12-month follow-up.

### Data analyses

We will assess the similarity of baseline characteristics between study arms using t-tests, chi-square tests, and Fisher's exact tests. We will control for any significant differences in baseline characteristics in analyses of all primary and secondary outcomes.

For each outcome, we will compare differences between study arms in 6-month changes using intent-to-treat analyses. Because this study is a quasi-experimental evaluation trial with a wait-list variant of a stepped wedge design, analyses of outcome changes will be conducted with two distinct methodologies. The first method will involve analyzing the data as if the trial was a standard intervention versus control study, comparing all participants who were randomized to the ATSM intervention (either SMARTSteps-ONLY or SMARTSteps-PLUS) versus all participants randomized to the wait-list control groups. We will use linear regression for continuous variables, logistic regression for dichotomous variables, and negative binomial models for the outcome variable of number of bed days because of its non-parametric data distribution. We will include as model covariates the baseline value of outcome variables and any socio-demographic or medical variables that may have differed by chance among the groups.

The second method will take advantage of the wait-list variant, stepped wedge design with study cross-over of participants from control to intervention [40]. It will employ a repeated measures analysis using maximum likelihood to fit the models. Because discrete external events may coincide with the time that participants cross-over from control to intervention and thereby possibly confound the within-group comparisons from the wait-list control group, secular effects in the wait-list group will be modeled first to see if a specific spline to capture non-linearities analysis is needed or if a simple linear trend exists, simplifying the analysis. Other possible covariates in the model may be calendar time, actual time of intervention exposure, and predictors of dropping out of study.

To explore the added value of the medication activation arm, both the first and second methods above will also be used to compare SMARTSteps-ONLY versus SMARTSteps-PLUS in all primary and secondary outcomes.

#### Sample size calculations

Sample size calculations were based on comparison of primary outcomes for the combined intervention groups (SMARTSteps-ONLY and SMARTSteps-PLUS) with the combined wait-list control groups. Based on an eligible population of approximately 500 SFHP members, we anticipated recruitment of 260 participants to intervention and control with a 10% drop-out or loss to follow-up rate at 6 months. Using a two-tailed test, we calculated that we would have 80% power to detect a standardized effect size (SE) of 0.35 in the primary outcome of SF-12 scores, comparing the combined intervention groups with the combined wait-list groups. In the prior RCT, we observed standardized effect sizes in this range [33].

We estimated that over the course of the two-year observation period, approximately 20% of enrolled participants would have insufficient data for secondary cardiometabolic outcomes to be included in these analyses. Under this assumption, we would have 80% power to detect a difference in HbA1c of 0.51% (SE 0.28) when comparing wait-list vs. combined ATSM interventions participants. This effect size is intermediate between that estimated to be the effect on glycemic control of diabetes disease management programs that involve team changes (-0.3% HbA1c improvement) and those that involve medication intensification strategies (-0.6%) [57].

### Recruitment

**Figure 3:**
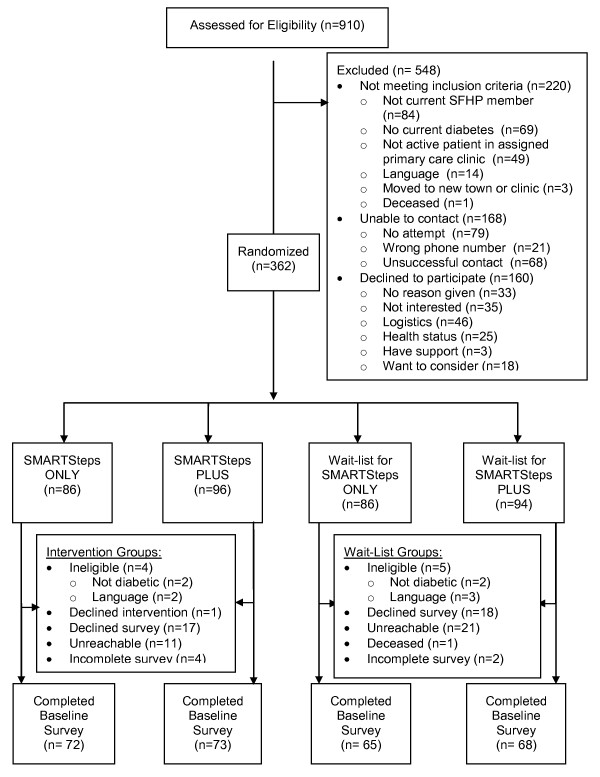
**Recruitment for SMARTSteps, a quasi-experimental evaluation trial of a language-concordant automated telephone diabetes self-management health plan intervention**.

Figure 3 shows the CONSORT diagram for recruitment of participants into the SMARTSteps Project by SFHP and into the UCSF evaluation. Of 910 SFHP members originally assessed for eligibility, 548 were excluded: 220 did not meet inclusion criteria, 168 could not be contacted, and 160 declined participation. Of note, because SFHP membership was continually changing, SFHP categorized people as "not eligible for SFHP" if they had not been an active SFHP member for more than a month and were not yet contacted by SFHP for recruitment.

At close of evaluation trial, 362 patients were enrolled and randomized for the SMARTSteps program, with 278 completing baseline interviews for the evaluation trial. Table 2 shows the socio-demographic and medical characteristics for those who enrolled in SMARTSteps, SFHP members who declined enrollment, and those who were not contacted for recruitment (some of whom may have been found ineligible if they had been contacted to complete the eligibility screening survey). Compared with the members who declined, SMARTSteps participants were younger; more likely to be women, Hispanic/Latino, and non-English-speaking; and less likely to be white/Caucasian. Compared with non-contacted members, SMARTSteps participants had LDL values and were more likely to be non-English speaking and to have Healthy Workers insurance.

**Table 2:** Baseline characteristics in SMARTSteps, a quasi-experimental evaluation trial of a language-concordant automated telephone diabetes self-management health plan intervention

Characteristic	Enrolled (N = 362)	Declined (N = 160)	p-value	Non-Contacted (N = 168)	p-value
Age in years, mean +/- SD	54.8 (8.4)	56.2 (9.2)	0.03	54.5 (10.9)	0.93

Female, n (%)	258 (71.3)	98 (61.3)	0.02	111 (66.1)	0.23

Race/ethnicity, n (%)			< 0.01		0.10
Asian	212 (58.6)	97 (60.6)		84 (50.0)	
Black/African-American	25 (6.9)	9 (5.6)		18 (10.7)	
White/Caucasian	34 (9.4)	31 (19.4)		20 (11.9)	
Latino/Hispanic	81 (22.4)	16 (10.0)		38 (22.6)	
Native American/Eskimo	1 (0.3)	0 (0.0)		2 (1.2)	
Hawaiian/Pacific Islander	3 (0.8)	0 (0.0)		0 (0.0)	
Other	5 (1.4)	6 (3.8)		5 (3.0)	
Unknown	1 (0.3)	1 (0.6)		1 (0.6)	

Language, n (%)			< 0.01		< 0.01
English	121 (33.4)	81 (50.6)		95 (56.5)	
Spanish	61 (16.9)	6 (3.8)		22 (13.1)	
Cantonese	180 (49.7)	73 (45.6)		51 (30.4)	

Financial Class - Insurance Type, n (%)			0.83		0.04
Healthy Worker	255 (70.6)	112 (70.0)		95 (56.5)	
Medicaid	82 (22.7)	35 (21.9)		53 (31.6)	
Medicare	16 (4.4)	9 (5.6)		10 (6.0)	
Healthy San Francisco	5 (1.4)	2 (1.3)		6 (3.6)	
Uninsured	3 (0.8)	1 (0.6)		3 (1.8)	
Commercial	0 (0.0)	1 (0.6)		1 (0.6)	
Healthy Kids	1 (0.3)	0 (0.0)		0 (0.0)	

Cardiometabolic Indicators, mean +/- SD					
Hemoglobin A1c	7.7 (1.6)	7.6 (1.5)	0.09	7.9 (1.9)	0.82
Systolic blood pressure	128.6 (17.6)	128.8 (16.7)	0.80	131.6 (19.2)	0.38
Diastolic blood pressure	74.7 (11.2)	75.2 (11.0)	0.48	75.6 (10.2)	0.31
Low-density lipoprotein	95.0 (30.6)	95.0 (34.3)	0.99	105.2 (34.0)	< 0.01

* All p-values were derived from chi-square tests for categorical variables, t-tests for interval variables if normally distributed and Wilcoxon tests if interval variables not normally distributed (age and cardiometabolic indicators).

† P-values for race/ethnicity were calculated based on categories of Asian/Pacific Islander, Black/African-American, Hispanic/Latino, Native American/Other/Unknown, and White/Caucasian.

‡P-values for financial class were calculated based on categories of Healthy Worker/Healthy San Francisco, Medicaid (Community Alternatives Program or Fee-For-Service), Medicare, and Commercial/Uninsured.

With SF-12 scores on 278 patients at baseline, assuming 10% loss to a 6-month follow-up sample of 250, we would have an 80% power to detect a mean difference of 2.85 in the SF-12 physical component score and 3.44 in the SF-12 mental component score.

Clinical registry data included available baseline HbA1c values for 348 total patients. With an assumption of 20% loss to follow-up, our study would have 80% power to detect a HbA1c difference of 0.37%.

## Discussion

The results of our recruitment process suggest that ATSM with health coach counseling may be a viable strategy for low-income managed care plans caring for linguistically and culturally diverse vulnerable persons with diabetes using population-based recruitment. In the IDEALL study, we found high levels of engagement, particularly with non-English speakers, and our early measures of reach suggest that this intervention model can be scaled up for larger populations [32,33,37].

The quasi-experimental design of the SMARTSteps evaluation trial offers a useful illustration of the advantages and challenges of a wait-list variant of a stepped wedge design. This design enabled all eligible SFHP members to receive ATSM as a benefit and increased the feasibility of scaling up the intervention through recruitment waves, while retaining elements of randomization that help minimize bias. However, this design requires careful evaluation of both intervention fidelity and potential confounders over time as well as investigation into the impact of extended follow-up time, potential time trends, and the impact of clustering in the data analysis strategy [40].

Translational evidence from safety net settings is needed to understand whether HIT applications such as ATSM can be effective in real-world settings and to inform how to harness HIT to support chronic disease self-management among vulnerable populations and improve patient-centered outcomes. In response to Medicaid and Medicare incentives arising from the 2009 Health Information Technology for Economic and Clinical Health Act, safety net settings are required to integrate electronic health records (EHRs) into the care environment and meet "meaningful use" criteria, including patient education and tracking of quality indicators [58-60]. In addition, federal and state policy has steered increasing numbers of vulnerable populations into capitated, managed care arrangements, with some promising results with respect to chronic disease outcomes and disparities reductions [61]. Expanding HIT integration among Medicaid managed health plans, provider groups, and patients will increasingly be a critical avenue for improving the health of vulnerable populations.

The results of this evaluation trial, when completed, must be interpreted within the following limitations. First, SFHP's phone-based population recruitment targeted different languages over time due to enrollment worker availability, with greater emphasis on Cantonese-speaking populations earlier. Thus, potential differences in intervention impact by language may relate to changes in project implementation over time. Second, because SFHP does not have medication prescribing authority for its members or authority to document within participants' medical records, the interventions will not be fully-integrated into care delivery in the context of participants' medical homes; this may limit the effectiveness of the intervention, particularly the SMARTSteps-PLUS medication intensification arm. Finally, because data for cardiometabolic outcomes relies on collection through routine medical care rather than scheduled study visits, we may not be powered to detect a modest impact of the intervention on glycemic or blood pressure control.

Improving health communication through the use of tailored, proactive HIT is one scalable vehicle to improve chronic disease care for vulnerable populations [32,33]. Focusing on improving the processes of care for vulnerable populations in under-resourced safety net settings through the application of evidence-based or evidence-informed HIT tools can have far-reaching implications for bridging the clinical care-public health divide, not only for diabetes, but for other chronic conditions and for preventive services [27]. The SMARTSteps outcomes will provide evidence regarding the real-world implementation of ATSM within a Medicaid managed care plan and provide insight into translating and scaling up HIT interventions for linguistically and culturally diverse vulnerable populations with chronic disease.

## List of Abbreviations Used

ATSM: automated telephone self-management; CATI: computer-assisted telephone interview; CHNSF: Community Health Network of San Francisco; HbA1c: hemoglobin A1c; HIT: health information technology; IPC: Interpersonal Processes of Care instrument; LDL: low-density lipoprotein; PACIC: Patient Assessment of Care for Chronic Conditions instrument; PCP: primary care provider; SDSCA: Summary of Diabetes Self-Care Activities instrument; SE: standardized effect size; SFHP: San Francisco Health Plan; SMARTSteps: Self-Management Automated and Real-Time Telephonic Support; UCSF: University of California, San Francisco

## Competing interests

The salaries of the investigators (MH, JQ, and MR) working on this study were partially funded by the McKesson Foundation. No funders had any role in the study design; collection, analysis, and interpretation of data; writing of the manuscript; or decision to submit the manuscript for publication.

## Authors' contributions

NR participated in the design and implementation of the study and drafted the manuscript. MH participated in the design and implementation of the study and helped to draft the manuscript. JQ participated in the implementation of the study, performed statistical analyses, and helped to draft the manuscript. US participated in the design and implementation of the study and helped to draft the manuscript. CS participated in the design and implementation of the study. KP participated in the design and implementation of the study. DS conceived of the study, oversaw the design and implementation of study, and helped to draft the manuscript. All authors read and approved the final manuscript.

## Pre-publication history

The pre-publication history for this paper can be accessed here:

http://www.biomedcentral.com/1472-6963/12/22/prepub

## Supplementary Material

Additional file 1 **SMARTSteps-PLUS medication intensification scripts**. This document offers an example of SMARTSteps-PLUS medication intensification scripts for health coach phone calls in a quasi-experimental evaluation trial of a language-concordant automated telephone diabetes self-management health plan intervention. Click here for file
