# Supplementary material for: Quasi-experimental trial of diabetes Self-Management Automated and Real-Time Telephonic Support (SMARTSteps) in a Medicaid managed care plan: study protocol
Source: BMC Health Serv Res. 2012 Jan 26;12:22. doi: 10.1186/1472-6963-12-22 (PMC3276419; doi:10.1186/1472-6963-12-22)
Supplement: Additional file 1 — SMARTSteps-PLUS medication intensification scripts. This document offers an example of SMARTSteps-PLUS medication intensification scripts for health coach phone calls in a quasi-experimental evaluation trial of a language-concordant automated telephone diabetes self-management health plan intervention. [file 1472-6963-12-22-S1.DOC]

Additional File 1: SMARTSteps-PLUS medication intensification scripts for health coach phone calls in a quasi-experimental evaluation trial of a language-concordant automated telephone diabetes self-management health plan intervention

**WHEN ASKING ABOUT INCREASING OR ADDING A MEDICATION:** Don’t make it sound like this should happen or that it will happen. *“I am just wondering because sometimes this is what your doctor would recommend. Your doctor knows we are asking his/her patients, so we can let him/her know which patients are interested in talking about this. If you are interested, I would just let your doctor know this.”*

- **If they say yes:** *“OK – I will let your doctor know. Don’t make any changes now before you talk to him/her again.”*
- **If they say no, say** *“OK – that’s fine. Tell me more about your reasons.”*  **Mark on screen.**
- **If they say, “I’d rather just talk to the doctor myself about it.”** You can respond: *“That's wonderful that you want to talk to your doctor. Your doctor actually wanted us to find out who wants to talk to him/her about this. We just want to let your doctor know you're interested in talking about this. Would you like us to do that?*

**General Communication Strategies**

- **Nonjudgmental**
- **OARS:**
  - **Open-ended questions:** *“What makes it hard to take your medications?” “Tell me about your diet.”*
  - **Affirmation:** *“You’ve done a great job taking your sugars.”*
  - **Reflective listening:** *“It sounds like you feel frustrated.”*
  - **Summaries:** *“Let me see if I understand. You feel too tired to exercise, and at the same time, you think you’d feel better if you lost weight. Is that right?”*
- **Teach back**

| **What are goals for good health in diabetes?** | Check knowledge about blood pressure | - *Tell me what you know about your blood pressure and how it relates to your diabetes.* - *Tell me what your blood pressure was like at your last clinic visit.* - *Do you check your blood pressure at home or at your neighborhood stores? Tell me what those are like.* - *Have you and your doctor talked about what your blood pressure should be?* |
| --- | --- | --- |
|  | Educate about goals | *The top number of blood pressure should be below 130. The bottom number should be below 80. This is important to protect your heart, brain, and kidneys. Good blood pressure helps prevent heart attacks, strokes, and kidney damage.* |
| **Introduction** | Check accuracy of reason for call (some pts have more than 1 reason) | **ATSM call:**  *In this week’s call, there were questions about the blood pressure medications you take. You mentioned you took your blood pressure medications “x” days this week. Does that sound right?* |
|  |  | **BP high***: At your last visit, your blood pressure was ___ / ___. This is high. Does that sound right?*  **BP missing:** It doesn’t look like you’ve had your blood pressure checked in a while. *Does that sound right?* |
|  |  | **Pharmacy claims:** *It looks like you haven’t picked up your ______ in a while. Does that sound right?* |
|  | Permissive statement | *Many patients take their medications differently from the way they are prescribed. As part of your care team, it’s important for me to understand how you’re taking your medications.* |
| **Check knowledge about current medications** | Open-ended | *What blood pressure medications are you taking right now? For each one, tell me their names, how much you take, and what they are for?*  (can ask patient to get bottles) |
|  | If can’t say: | *Are you taking a medication called ___? Tell me how you’re taking it.* |
|  | If different from rx: | *I just want to check about ___. Your doctor thinks you are taking* (name of med*) ___ times per day. That seems different from what you just said. Can you tell me more about that?* |
| **Check for other barriers to adherence – ask all** | Side effects | *Tell me about any side effects or problems you have when you take* [insert names]. (Address any questions, and work with patient to problem solve or suggest talking to PCP.) |
|  | Health beliefs | *Do you sometimes feel you don’t need to take as much of these medications? What are your worries or concerns about these medicines?* (Be nonjudgmental, and then address any misconceptions. If resistant, encourage them to talk to PCP.) |
|  | Other barriers | *What makes it hard for you to take your blood pressure medications?*   - Able to get medications - Able to pay for medications - Remembering to take medications - Remembering to pick up refills |
| **IF ADHERENCE ISSUES:** | | |
| **Make an adherence action plan** | Transition | *It’s very important to figure out better ways to take your medications. Let’s work on a plan together* |
|  | Member-generated | *Tell me something you’d like to change about how you take your blood pressure medications.* |
|  | Highly specific | - *WHAT small step do you think you can take to start?* - *HOW MUCH of that do you think you can do next week?* - *WHEN would be the best time of day for you to do this?* - *How many days during the next week do you think you can do this?* |
|  | Achievable | *On a scale of 0-10, with 0 being totally unsure and 10 being totally sure, how sure are you that you can do [action plan] next week?* **(if numbers hard, could use “very sure, pretty sure, a little sure, not at all sure”)**  **(if <7 or “a little / not at all sure,” help them re-do plan)** |
|  | If no ideas, offer options but let member pick what works for them. | - - - Link medication use (in location and time) to existing routine (e.g. remote control, coffee, tooth brushing)     - Use cues and reminder aids (e.g. Post-Its, calendars, alarms)     - Family / friend participation     - Pharmacy help +/- mediset |
| **IF NO ADHERENCE ISSUES:** | | |
| **Make an Action Plan** | Transition | *It’s very important that you and your diabetes health care team work together to lower your blood pressure.* |
|  | Member-generated | *Tell me something you’d like to change about your blood pressure.* |
|  | Highly specific | - *WHAT small step do you think you can take to start?* - *Great. HOW MUCH of that do you think you can do during the next week?* - *WHEN would be the best time of day for you to do this?* - *How many days during the next week do you think you can do this?* |
|  | Achievable | *On a scale of 0-10, with 0 being totally unsure and 10 being totally sure, how sure are you that you can do [action plan] next week?* **(if numbers hard, could use “very sure, pretty sure, a little sure, not at all sure”)**  **(if <7 or “a little / not at all sure,” help them re-do plan)** |
| **Assess willingness to intensify** | Increase dose | *If your diabetes health team were to recommend that you TAKE MORE of your current medication, would you agree to do so? Why or why not?* |
|  | New medication | *If your diabetes health team were to recommend that you take a NEW medication, would you agree? Why or why not?* |
| **RESUME ALL:** | | |
| **Concluding Call** | Teach back | *Great. It would help me if you could tell me your action plan in your own words.* (**If not clear, re-educate and repeat teach back.)** |
|  | Reinforcement | *It is very important to take care of your health / take your medicines so you can [feel better / stay healthy and active for a long time].*  *It’s also important for your diabetes care team to know how you feel about your medications.* |
|  | Next steps | *We will tell your diabetes health care team about our phone call.* |
|  | **If safety concern** | *You should call the clinic to set up a visit or talk to you more by phone.* |
